# Expression of Recombinant Streptokinase from *Streptococcus Pyogenes and* its Reaction with Infected Human and Murine Sera

**Published:** 2013-09

**Authors:** Neda Molaee, Hamid Abtahi, Ghasem Mosayebi

**Affiliations:** 1 Department of Cellular and Molecular Biology, Science and Research Branch, Islamic Azad University, Tehran, Iran; 2 Molecular and Medicine Research Center, Arak University of Medical Sciences, Arak, Iran

**Keywords:** Anti-streptokinase, Gene expression, Recombinant, Streptokinase protein, *Streptococcus pyogenes*

## Abstract

***Objective(s): ***Streptokinase (SKa) is an antigenic protein which is secreted by *Streptococcus pyogenes*. Streptokinase induces inflammation by complement activation, which may play a role in post infectious diseases. In the present study, recombinant streptokinase from *S. pyogenes* was produced and showed that recombinant SKa protein was recognized by infected human sera using Western blot analysis.

***Materials and Methods:*** In this study, the *ska* gene from *S. pyogenes* was amplified and cloned into pET32a which is a prokaryotic expression vector. pET32a-ska was transformed to *Escherichia coli* BL21 (DE3) pLysS and gene expression was induced by IPTG. Protein production was improved by modification of composition of the bacterial culture media and altering the induction time by IPTG. The expressed protein was purified by affinity chromatography using the Ni-NTA resin. The integrity of the product was confirmed by Westernblot analysis using infected mice. Serum reactivity of five infected individuals was further analyzed against the recombinant SKa protein.

***Results:*** Data indicated that recombinant SKa protein from *S. pyogenes* can be recognized by patient and mice sera. The concentration of the purified recombinant protein was 3.2 mg/L of initial culture. The highest amount of the expressed protein after addition of IPTG was obtained in a bacterial culture without glucose with the culture optical density of 0.8 (OD_600_ = 0.8).

***Conclusion*** *:* Present data shows, recombinant SKa protein has same epitopes with natural form of this antigen. Recombinant SKa also seemed to be a promising antigen for the serologic diagnosis of *S. pyogenes *infections.

## Introduction

Group A streptococcus (*Streptococcus pyogenes*, or *GAS*) is a β-hemolytic *Streptococcus* which is responsible for most cases of streptococcal illness, ranging from mild localized infections to severe disseminated disease. The variety of diseases is due to the virulence factors that either exist on the surface of the bacterium or are secreted by the organism. One of the secreted factors shown to be important for streptococcal virulence is streptokinase, that is encoded by the *ska* gene (1).

Streptokinase is a plasminogen activator agent that can be secreted by group A, C, and G streptococci. Streptokinase contributes to streptococcal virulence by generating plasmin, which leads to bacterial spread from a primary location of infection by causing fibrinolysis and degradation of extracellular matrix and basement membrane components (2, 3). Streptokinase also induces inflammation by complement activation, which may play a role in post infectious diseases, such as glomerulonephritis (4).

Comparison of the nucleotide sequence of the DNA fragment carrying SKa from group A and group C streptokinase gene (*skc*) demonstrated 90% sequence identity between the two genes, with highly conserved transcription and translation control regions. The deduced amino acid sequence of the *S. pyogenes* contains the same number of amino acids as that of group C streptokinase, with 85% sequence identity (5, 6).

Diagnosis of* S. pyogenes *infection must be confirmed by culture and serological test. However, the result of the culture is worthless after about two to three weeks after initial infection. Therefore, detection of the *S. pyogenes *antibody is used to determine if a streptococcal infection is present (7, 8).

Serological diagnosis of group A streptococcal infections is based on immune responses against the extracellular enzymes streptolysin O, streptodornase B, hyaluronidase, DNase, and streptokinase. These enzymes induce strong immune responses in infected host (8, 9). In the present study, recombinant streptokinase from S*. pyogenes* was produced and shown that recombinant SKa protein was recognized by infected human sera using Western blot analysis.

## Material and Methods

***Bacterial strains and plasmids***

*S. p*yogenes (ATCC: 8668) was used as the source of chromosomal DNA for the PCR (polymerase chain reaction). *E. coli* DH5α (Stratagene) was used as the primary host for the construction and propagation of plasmids. For recombinant protein production, a prokaryotic expression vector pET32a (Novagene) was selected. The recombinant pET32a (pET32a-SKa) is transformed in *E. coli*, BL21 (DE3) pLysS as host strain. LB agar (Merck Germany) and broth was used for routine bacterial culture. The required antibiotics were added to media according to references recommendation (10).

***Isolation of chromosomal DNA***

Chromosomal DNA was extracted and purified, as previously described (11).

***Gene amplification of streptokinase***

Primers were designed according to *S. pyogenes *streptokinase sequence (accession No. Z48617.1). The forward primer (GGGATCCATGAAAAATTACTT-

ATCT-3') starts from the beginning of the gene and contains *Bam*HI site on its 5` portion. Reverse primer, (5'- ACTCGAGTTATTTGTCTTTAGGGT -3') contains recognition site for *Xho*I. PCR was performed in a 50 μl total volume containing 500 ng of template DNA, 1 μM of each primers, 2.5 mM Mg^2+^, 200 μM (each) deoxynucleoside triphosphates, 1X PCR buffer and 2.5 unit of pwo DNA polymerase (Roche). The PCR started with an initial denaturation step at 94°C for 5 min (hot start), followed by thirty cycles of denaturation at 94°C for 1 min, annealing at 50°C for 1 min and extension at 72°C for 1 min. The program was followed by a final extension at 72°C for 5 min.

The PCR product was sequenced by MWG Company (Germany) to confirm that the desired product had been obtained.

***Cloning of SK gene into the expression vector pET32a***

The PCR product and pET32a were digested with *Bam*HI and *Xho*I and ligated into pET32a by T4 DNA ligase (Cinagen, Iran) at 16°C overnight. The competent cells of *E. coli* DH5α and *E. coli* BL21 (DE3) plysS were prepared by calcium chloride method and were used for transformation of pET32a-SKa plasmid (12).

***Expression and purification of recombinant SKa***

For protein expression, BL21pLysS cells were transformed with pET32a-Ska construct. Bacterial cells were grown in LB broth supplemented with ampicillin (100 μg ml-1) and chloramphenicol (34 μg ml-1) at 37°C with agitation. The protein induction was performed in the two media using IPTG (Isopropyl-β-D-thio-galactoside, Cinagene). IPTG was added to different concentration of bacteria (OD_600nm_ 0.6, 0.8, and 1) in both culture media. After 2 and 4 hours of induction, bacterial cells were collected by centrifugation at 1700 g for 15 min. A 15% SDS-PAGE system was used to analyze the resulting proteins. The expressed protein was purified using Ni-NTA column according to manufacture instructions (Qiagene, USA). The purified protein was dialyzed twice against PBS (pH 7.2) at 4°C over night. The quantity of purified recombinant Streptokinase protein was analyzed by Bradford methods and subsequently its quality was assayed by SDS-PAGE 15% (11, 13).

***Immunization of mice***

The supernatant of *S. pyogenes* culture were centrifuged at 1700 g for 5 min and filtered using a 0.42 µM disposable syringe filter (Milex, USA). The resulted emulsion and equal volume of complete Freund’s adjuvant were injected subcutaneously to five mice at 3 weeks intervals. Mice sera were separated and used as primary antibody.

In addition, five acute phase patients' sera were obtained from Immunology Department (Arak University of Medical Sciences, Arak, Iran) to be used in Western blot experiment.

***Antigenicity of recombinant streptokinase and Immunoblot analysis***

Western blot assay was done using 0.5 μg of purified recombinant SKa protein per well. The gel were blotted on to polyvinylidine difluoride (PVDF membrane, Roche, Germany) membrane using transfer buffer containing 25 mM Tris (pH = 8.3), 192 mM glycine and 20% methanol at 90 volts for 1.5 hr at 4°C. The blotted membrane was blocked with 2.5% (w/v) BSA in TBST buffer (0.5 M NaCl, 0.02 M Tris pH 8.5, 0.05% tween 20) for 1 hr at room temperature. After reactions with the primary antibody (mice and patients sera, diluted 1:100 and 1:50, respectively), the blots were washed three times with TBST and incubated with peroxidase conjugated goat anti-mouse IgG and antihuman IgG (sigma) at a 1:2500 dilution in TBST.

The blots were then washed three times with TBST and reactions were developed by diamino banzidine (DAB) solution (Roche) (10).

**Table 1:** Different concentrations of culture media

Medium content	Media-1	Media-2
Yeast extract (g)	0.25	0.25
Bacto tryptone broth (g)	0.5	0.5
glucose (g)	_	0.05
NaCl (g)	0.25	0.25
KCl (g)	0.025	0.025
MgCl2 (g)	0.013	0.013
Cacl2 (g)	0.013	0.013
Final volume	25 ml	25 ml

## Results

***PCR product analysis***

The sequencing result was confirmed by comparing the NCBI nucleotide database using basic local alignment search tool (BLAST) Software (data not shown). The sequencing of the PCR product fragment revealed complete homology at the nucleotide level to *ska* gene in NCBI (GenBank: Z48617.1).

***Expression and purification of recombinant SKa***

**Figure 1:**
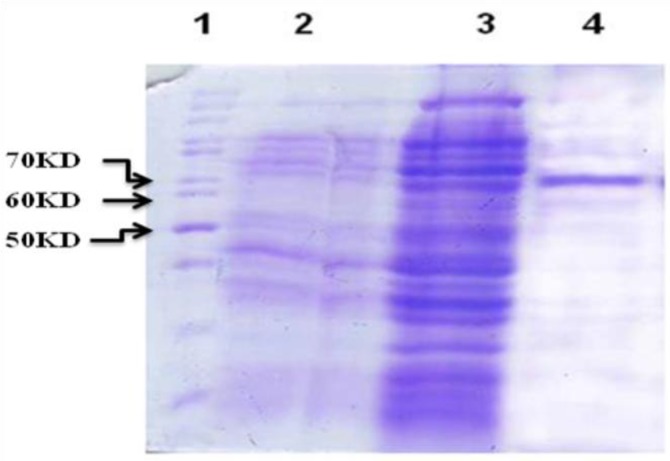
Gel protein electrophoresis, expression of recombinant *SKa* protein and its purification. Lane 1, protein marker; Lane 2, before induction; Lane 3, after induction; Lane 4, elution of recombinant SKa protein through Ni-NTA column

In this study pET32a-SKA was transformed to BL21 (DE3) pLysS and the expressed protein was purified by Ni-NTA column (Figure 1). SDS-PAGE analysis, showed the expected molecular mass of near 65 kDa recombinant protein. The result also showed that the best conditions for recombinant SKa protein expression can be achieved when OD_600 nm_ = 0.8 is used for four hr. Production of recombinant protein was low in media with glucose. The concentration of recombinant protein was assayed and calculated to 3.2 mg purified protein per liter of the initial culture.

***Immunoblotting analysis***

**Figure 2:**
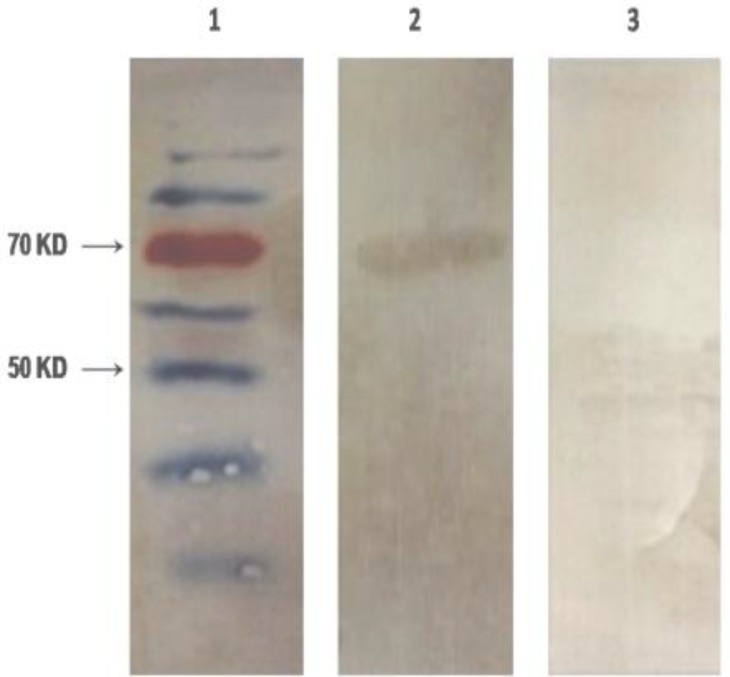
Western blot analysis against recombinant Streptokinase protein by mice sera. Lane 1, protein marker; Lane 2, Western blotting by mice infected sera; Lane 3,Western blotting by normal mouse serum (negative control

**Figure 3:**
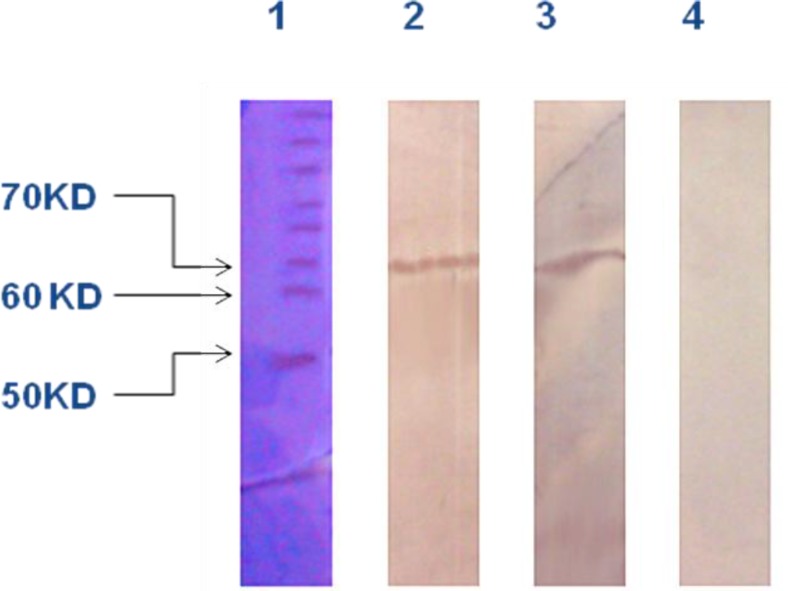
Western blot analysis against recombinant streptokinase protein by infected and normal human sera. Lane 1, protein marker; Lane 2 and 3, Western blotting by patients sera; Lane 4, human normal sera (negative control

To determine the antigenicity of recombinant streptokinase in patients and mice immunized with *S. pyogenes*, the recombinant streptokinase was assayed by Western blotting. The mice sera and five patients’ sera were used. Figure 2 illustrates the specific interaction between mice sera antibody and purified recombinant streptokinase protein. Due to observation of specific antibody responses from sera, antigenicity of the expressed product was confirmed, (Figure 2, lane 2 and 3). Serum sample from normal individual was also tested as negative control and no anti-streptokinase antibodies were detected, (Figure 3 lane 4).

## Discussion

In this study, we have shown that recombinant streptokinase can be detected by infected human sera.

The streptokinases are a family of secreted streptococcal proteins with the common function of converting plasminogen to plasmin (14). This fibrinolytic protein forms a 1:1 complex with plasminogen and converts other plasminogen molecules to plasmin. Streptokinase A is an antigenic protein that is naturally secreted by several strains of beta-hemolytic streptococci. Anti streptokinase antibody is able to neutralize the activity of streptokinase. The polymorphic regions of streptokinase span amino acid residues 174 to 244 and 270 to 290 (15). Despite the polymorphism in the variable regions of the molecule, the overall structure (hydrophilicity, hydrophobicity, antigenic sites, amphipathic regions, etc.) is maintained. Streptokinase activity found in different streptococci reflects the host range and is not active in hosts which it does not normally infect. Streptokinase has been associated with the pathogenesis of acute post streptococcal glomerulonephritis (15, 16).

**Table 2:** The amounts of produced protein at different induction times by IPTG, in the presence and absence of glucose

*1*	*0.8*	*0.6*	OD_600__nm_ =600	
*2.45 mg/l*	*2 mg/l*	*1.35 mg/l*	With Glucose	Media culture
*3 mg/l*	*3.2 mg/l*	*1.35 mg/l*	Without Glucose	

Diagnosis of *S. pyogenes *is based on isolation of streptococcus in culture or serological tests. Serological diagnosis of group A streptococcal infections is based on immune responses against the extracellular products streptolysin O, DNase B, hyaluronidase, NADase, and streptokinase, which induce strong immune responses in the infected host (17). Anti-streptolysin O (ASO) is the antibody response most often examined in serological tests to confirm antecedent streptococcal infection. The ASO titer is ordered primarily to determine whether a previous *S. pyogenes *infection has caused a post streptococcal disease, rheumatic fever or glomerulonephritis. Antibodies to streptolysin O are produced in approximately 75 to 80% of *S. pyogenes *infections, but are usually not seen in cutaneous infections caused by group A streptococcal infections. False positive results may occur from hypercholesterolemia in ASO test. If an anti-streptodornase B, antihyaluronidase or anti-streptokinase assay is performed on sera, the patient with at least one positive antistreptococcal enzyme titer that rises to 95% (18, 19).

Our data suggests that recombinant streptokinase of *S. pyogenes* can be produced by pET32a expressionin *E. coli*. The highest amount of the expressed protein after addition of IPTG was obtained when the optical density of the bacterial culture was OD_600_ = 0.8 and the culture did not have glucose.

Based on the obtained results, there was a low level of recombinant protein production when *E. coli* were grown in a culture containing glucose. It seems that in the presence of glucose, cyclic AMP levels becomes low, therefore, transcription from the *lac *promoter becomes low as well (glucose effect). This phenomenon is named catabolic repression and is shared by a number of *E. coli *operons. When glucose is absent and the cell is forced to use an alternative carbon source, such as glycerol, cAMP levels rise. The resulting formation of the CAP/cAMP complex stimulates transcription from the *lac *promoter. Therefore, full induction of the *lac *operon is achieved only in the presence of both inducer and elevated cAMP levels. In addition, in the presence of glucose, some byproduct metabolites is produced in the medium and these byproducts reduce the acidity of the bacterial culture and, therefore, bacterial cells reach stationary phase faster (20). It was also shown that the concentration of the bacterial cells has a fundamental effect on the protein expression. In other words, with increasing bacterial concentration up to OD_600_= 0.8, the efficiency of induction with IPTG was improved as well. But when the concentration of bacteria was increased more (OD_600_= 1), the induction efficiency was declined gradually. Therefore, the results of our study showed that in the presence of glucose in culture media and with the increase of bacterial concentration over the optical density of 0.8, the efficiency of recombinant protein production can be reduced.

Streptokinase, a secreted plasminogen-binding protein, is a single chain polypeptide with a molecular weight of 46 kDa. The coding sequence of *ska* gene is highly conserved among streptococcus species. There are two conserved domains separated by a more variable region (6). Our results indicate that the highly regulated expression vector with powerful T7 promoter (pET32a), in conjunction with suitable host cell [*E. coli, *BL21 (DE3) pLysS], could influence the rate of expression. The expressed protein from pET system contains several extra amino acids (6xHis tag and T7 tag) linked to the C or N terminal extension of the protein. These additional amino acids increase the size of expressed protein by approximately 20 kDa, as shown in Figure 1 (7).

## Conclusion

Present data shows that recombinant streptokinase can be detected as an antigen by serum in patients and immunized mouse. Therefore, recombinant SKa protein has same epitopes with natural form of this antigen. Recombinant SKa also seemed to be a promising antigen for the serologic diagnosis of *S. pyogenes *infections.
